# Transcriptomic evidence of a para-inflammatory state in the middle aged lumbar spinal cord

**DOI:** 10.1186/s12979-017-0091-6

**Published:** 2017-04-13

**Authors:** William Galbavy, Yong Lu, Martin Kaczocha, Michelino Puopolo, Lixin Liu, Mario J. Rebecchi

**Affiliations:** Department of Anesthesiology, School of Medicine, Health Sciences Center L4, Stony Brook University, Stony Brook, New York, 11794-8480 USA

**Keywords:** Transcriptome, Aging, Spinal cord, Microglia, Neuropathic, Complement, T-cell, Inflammation

## Abstract

**Background:**

We have previously reported elevated expression of multiple pro-inflammatory markers in the lumbar spinal cord (LSC) of middle-aged male rats compared to young adults suggesting a para-inflammatory state develops in the LSC by middle age, a time that in humans is associated with the greatest pain prevalence and persistence. The goal of the current study was to examine the transcriptome-wide gene expression differences between young and middle aged LSC.

**Methods:**

Young (3 month) and middle-aged (17 month) naïve Fisher 344 rats (*n* = 5 per group) were euthanized, perfused with heparinized saline, and the LSC were removed.

**Results:**

~70% of 31,000 coding sequences were detected. After normalization, ~ 1100 showed statistically significant differential expression. Of these genes, 353 middle-aged annotated genes differed by > 1.5 fold compared to the young group. Nearly 10% of these genes belonged to the microglial sensome. Analysis of this subset revealed that the principal age-related differential pathways populated are complement, pattern recognition receptors, OX40, and various T cell regulatory pathways consistent with microglial priming and T cell invasion and modulation. Many of these pathways substantially overlap those previously identified in studies of LSC of young animals with chronic inflammatory or neuropathic pain.

**Conclusions:**

Up-modulation of complement pathway, microglial priming and activation, and T cell/antigen-presenting cell communication in healthy middle-aged LSC was found. Taken together with our previous work, the results support our conclusion that an incipient or para-inflammatory state develops in the LSC in healthy middle-aged adults.

**Electronic supplementary material:**

The online version of this article (doi:10.1186/s12979-017-0091-6) contains supplementary material, which is available to authorized users.

## Background

Numerous studies have documented age-related inflammatory marker expression in the central nervous system (CNS), particularly of pro-inflammatory cytokines interleukin 1β (IL1β), tumor necrosis factor α (TNFα) and IL6, as well as microglial activation markers Cd11b (C3A receptor) and MHCII and the astrogliosis marker glial fibrillary acidic protein (GFAP) in various brain regions of healthy animals, including rats, mice, primates and in post-mortem human samples [1–4]. Challenging the senescent CNS with stimuli that mimic infection or stress, such as LPS, induce exaggerated neuroinflammatory responses [4, 5]. These results suggested a pre-existing incipient or para-inflammatory state that predisposes the CNS to deleterious neurotoxic response in older animals and humans, and have led to the idea that the CNS undergoes a process of “inflammaging”, with important implications for neurodegenerative disorders, such as Alzheimer’s dementia and Parkinson’s disease [2, 6, 7].

While the effects of aging on inflammatory marker expression in various brain regions are well established, few studies have examined the spinal cord, particularly of animals corresponding to middle-age, a time when chronic pain incidence and persistence reach a maximum in the human populations [8–13]. We have recently demonstrated that changes in expression patterns of inflammatory markers indicate that a para-inflammatory state in the lumbar spinal cords (LSC) arises by middle age in healthy rats [14]. This is accompanied by remarkable changes in dorsal horn microglial morphology rarely seen in young adult LSC, that indicated activated M1 and M2 morphologies (shortened, thickened processes, decreased arborization, and hypertrophic cell bodies). In the present study we compared the transcriptomic expression of whole lumbar spinal cords of healthy middle-aged male rats to those of young adults. The results provide further support for a para-inflammatory status in the LSC by middle age and point to development of microglial states previously associated with establishment of neuropathic or inflammatory pain.

## Methods

### Perfusion, tissue harvesting, RNA extraction and purification

All work conformed to the National Institutes of Health Guidelines for the Care and Use of Laboratory Animals and were approved by the Stony Brook University Institutional Animal Care and Use Committee and conducted under protocol #203692-23. Three-month and 17-month old Fisher 344 rats were were euthanized and transcardially perfused with heparinized saline-buffered with 5 mM H_2_NaPO_4_ to pH 7. LSC were rapidly removed and immediately frozen on dry ice and stored at -80 °C. Total RNAs were extracted from LSC using Qiazol extraction, and further purified with RNeasy spin columns following the manufacturer’s directions and as described previously [14]. Briefly, frozen tissues were placed on ice, Qiazol lysis reagent (Qiagen) was added immediately along with three or six 2.3 mm silica/zirconia beads (DRG and LSC, respectively), and homogenized in a BioSpec mini bead beater for 1.5 min and allowed to stand on ice for 5 min. Chloroform was added to comprise 1/5 of the total volume, and samples were mixed vigorously for 2 min and allowed to settle for 2 min before being centrifuged at 12,000 X g for 15 min at 4 °C. The upper aqueous phase was saved, mixed 1:1 with 70% ethanol, and subjected to RNeasy spin column purification (Qiagen). Final concentrations and 230/260/280 ratios were determined by nanodrop absorbancy using an Eppendorf BioSpectrometer.

### Transcriptomic analysis

The RNA’s were reversed transcribed, and then, using an in vitro transcription reaction, biotinylated nucleotides were incorporated, converting the cDNA to labeled cRNA. The cRNA pool was purified, fragmented and then hybridized to Rat Genome 230 2.0 arrays (Affymetrix) displaying over 31,000 probe sets, representing 30,000 transcripts and variants from over 28,000 well-substantiated rat genes. Hybridized chips were washed, incubated with fluorescently labeled streptavidin probe, laser scanned and probe fluorescence intensities were measured. Quality control analysis was performed using the affyQCReport package in Bioconductor. CEL files were quantified and normalized using GenePattern ExpressionFileCreator function at the setting of RMA method and quantile normalization. The relation of samples was displayed in dendrogram that was generated by hclust package in Bioconductor. Comparison of samples was conducted using R statistical project. The FDR values were calculated using the samr package in Bioconductor. The normalized data were then subjected to t-testing with FDR = 10%. Annotation of probe sets was based on Affymetrix Rat230_2.na34 release.

### qPCR Analysis

Primers were designed with Primer3 and synthesized by Eurofins MWG Operon LLC (Louisville, KY, USA). The primer pairs used in the qPCR reactions are listed in Additional file 1. cDNA was synthesized with QuantiTect Reverse Transcription Kit (Qiagen, Germantown, MD, USA) using the same mRNA that were used in the microarray measurements. qPCR analysis was performed on an Step-One Plus qPCR equipment (Applied BioSystems), using the Quantitect SYBR Green Kit (Qiagen). PCR reactions were followed by melt curve analysis. For data analysis, ΔC_t_ were acquired by subtracting the C_t_ values of the genes of interest from the C_t_ of a reference gene (GAPDH) of the corresponding samples. The ΔC_t_’s for the young and middle-aged groups were subjected to t-testing (one-tailed). Adjusted *p* value for multiple comparisons was carried out with R using the method of Benjamini and Hochberg [15].

### Pathway analysis

The differential expression gene set was subjected to Pathway analysis using IPA software suite. Three hundred fifty three differentially expressed, annotated genes that were increased or decreased significantly were imported and analyzed using Canonical pathways. Adjusted *p* values were obtained from a modified Fisher test [15] that compared the ratio of differential gene set to number of pathway members obtained to the probability that such a ratio would be found by chance, after correcting for multiple hypothesis testing.

## Results

**Fig. 1:**
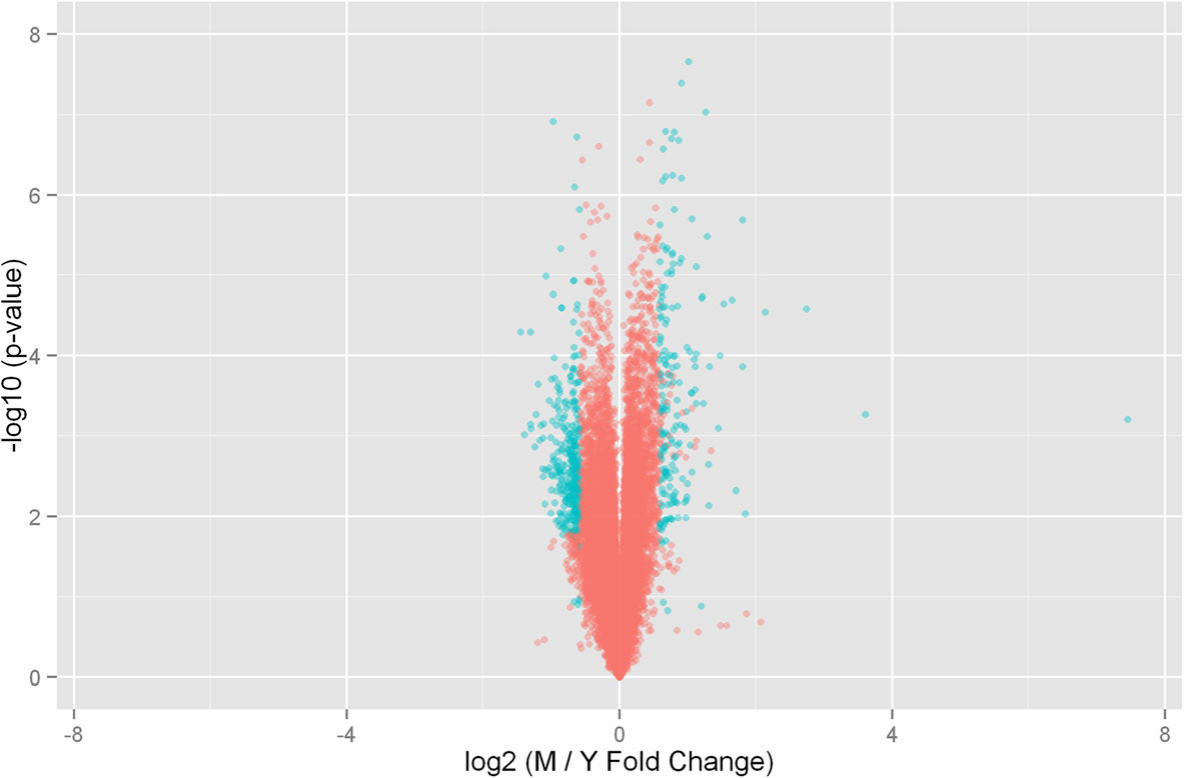
Transcript expression ratio’s in middle-aged compared to young LSC. The mRNA transcript expression in the LSC of 3 month and 17-month-old Fisher 344 male rats were assessed and 21,700 probe sets were measureable. The *horizontal axis* indicates the log_2_ ratios of middle aged over young expression and the *vertical axis* is the –log_10_ of the FDR corrected *p* values. ~ 1100 transcripts were differentially expressed. *Blue dots* represent genes that increased or decreased by at least 1.5 fold (528)

Transcriptomic expression patterns were determined in LSC from young (3 month) and middle-aged (17 month) male Fisher 344 rats. Of the 28,000 genes probed, over 1,100 showed significant differential expression (Fig. 1). Five hundred twenty eight genes increased or decreased at least 1.5 fold in middle age (Additional file 2). Of these, 353 unique well-annotated genes were subjected to further analyses (Additional file 3). Table 1 lists annotated genes with the greatest differential expression. The largest change associated with middle age was expression of Dnajb12 (6.70 fold increase). Dnajb12 and related genes regulate proteasomal degradation of polytopic membrane proteins, particularly ion channels [16]. Gipr transcript, which encodes the receptor for gastric inhibitory polypeptide (GIP), also substantially increased. Agonist analogs of GIP reduce central oxidative stress, and are neuroprotective in Alzheimer’s disease [17, 18], and stroke models [19]. Gpnmb transcript levels were elevated over three fold. This mRNA encodes a regulator of immune responses expressed in microglia that appears to have anti-inflammatory properties [20]. Among transcripts reduced in middle age, Herc1 participates in membrane trafficking; its loss increases autophagy and decreases mTOR activity associated with Purkinje cell degeneration [21]. Kif1a expression, which is required for BDNF-induced synaptogenesis in the hippocampus [22], was also significantly diminished in middle age.

**Table 1:** Differential gene expression: largest effect sizes

Gene	M/Y	Y Mean	M Mean	Annotation
Abp10	0.46	677	311	Annexin V-binding protein
Aff4	0.45	458	204	Transcription factor and central SEC component
Ago2	0.43	330	141	RISC Catalytic Component 2
Aqp4	0.50	2664	1329	Aquaporin family member
Arl11	2.07	94	195	ADP-ribosylation factor-like 11
Asap1	0.40	524	212	ADP-ribosylation factor (ARF) GTPase-activating protein
Bmpr2	0.46	970	449	Bone morphogenetic protein receptor type II
C3	2.40	830	1996	Central complement component
Chrdl1	2.48	60	148	Ventroptin, antagonizes BMP actions
Clec12a	2.18	99	215	CTL/CTLD member, widely expressed in innate immune system
Cspp1	0.49	207	101	Important for normal neural specific cilia function
Dnajb12	6.70	52	350	DNAJ/HSP40 family member, regulates molecular chaperone activity, protein folding and degradation
Enc1	0.47	135	64	Kelch-related family of actin-binding proteins, role in oxidative stress response, regulates Nrf2
Falz	0.50	482	239	Transcription/epigenetic regulator, up-modulated in neurodegenerative diseases
Fcgr2b	2.89	303	878	Low affinity Fc receptor expressed in microglia
Fcrls	2.78	218	607	Fc receptor; part of TGFb1 microglial signature response
Fmo2	2.35	87	204	Flavin-containing monooxygenase
Fstl3	2.13	66	142	Secreted form binds/antagonizes members of TGF-b family, e.g., BMP2
Gipr	3.49	179	623	Receptor for neuroprotective polypeptide GIP
Golga4	0.42	211	89	Role in Rab6-regulated membrane-tethering events in the Golgi
Gpnmb	3.14	420	1317	Expressed in microglia, inflammatory response gene
Herc1	0.36	294	107	Regulator of membrane trafficking
Kif1a	0.38	3058	1160	Kinesin family protein involved in syaptogenesis
Lgals3	2.44	927	2264	Member galectin family of carbohydrate binding proteins; microglial marker and alt priming gene
Msi2	0.49	304	148	RNA binding protein; role in CNS stem cell proliferation
Nrg1	0.46	553	253	Neuregulin-1, signals through erb2/3 receptors, functions in neural development and plasticity
Pitpnm1	3.50	499	1749	Transfers PtdIns from ER to plasma membrane
Postn	2.73	147	402	Aka periostin; enhances TGF-β signaling, facilitates BMP1, expressed in reactive microglia and astrocytes, neuroprotective
Pou3f1	2.16	266	574	Transcription factor, promotes stem cell commitment to neural fate
RT1-EC2	2.50	75	189	Class I MHC, up-modulated in spinal cord injury and neurodegeneration
S100a8	2.30	85	194	Induced in macrophages and dendritic cells by TLR agonists, oxidative stress and corticosterone
Slc9a3	2.15	195	421	Sodium/proton exchanger; Cation Proton Antiporter 3
Tnc	2.32	75	173	Extracellular matrix protein, up regulated following CNS trauma
Unc5b	0.47	995	469	Netrin receptor family; required for axon guidance; maintains blood–brain barrier
Usp7	0.46	121	56	Uiquitin-specific-processing protease 7, deubiquitinates range Deubiquitinates target proteins

The annotated differential gene set (353) was filtered for largest effect sizes. Mean probe set expression levels (arbitrary units) for Y (young) and M (middle age) genes are shown

To confirm the reproducibility and accuracy of the microarray measurements, we performed qPCR analyses on the same sets of samples measuring the relative change in expression (normalized to GAPDH mRNA). Our results (Table 2) show a close correspondence between qPCR and microarray results for 8 differentially expressed genes: Lgals, Fcr2b, GPNMB, C3, Atf3, Ptprc, Cd163 and Nrg1, as well as induction of the microglial activation marker Cd11b.

**Table 2:** Confirmation of microarray results by qPCR

Results	Lgals	Fcgr2b	Gpnmb	C3	Atf3	Ptprc	Cd163	Nrg1	Cd11b
ΔΔC_t_ M v Y	1.62	1.68	1.76	1.42	0.94	0.99	0.78	−0.79	0.87
qPCR Fold M/Y	3.08	3.20	3.40	2.68	1.92	1.99	1.72	0.58	1.83
Microarray M/Y	2.44	2.89	3.14	2.40	1.73	1.84	1.59	0.46	N/A
Adjusted *p*-value	0.004	0.003	0.006	0.010	0.004	0.001	0.001	0.056	1.0E-07

cDNA was synthesized from the same RNA samples analyzed in the microarrays. ΔΔC_t_ values are expressed as described in Methods

The annotated differential gene set consisting of the 353 transcripts, was subjected to pathway analysis (IPA) using its curated pathway database. Of 13 canonical pathways, complement cascade had the highest proportion of differentially expressed genes and lowest adjusted *p* value (Table 3). In the complement system, 9 differentially expressed aged-related genes were up modulated out of 37 Complement system members (0.243; *p* = 1.11 × 10^−6^). Members of the Classical pathway, initiated by C1q activation and the Alternative pathway, initiated by C3 activation were well populated. The positive Z-score = 1.414, indicated significant complement pathway activation. On the other hand, expression of several inhibitory components, Serping1, a C1 esterase inhibitor, and Cfh, which accelerates C3b inactivation, were increased in middle age (1.55 and 1.79 fold, respectively), and could possible suppress these complement activation [23].

**Table 3:** Canonical pathways populated by the age-related gene set

Pathway	Target Genes	Pathway Genes	Ratio	z-score	B-H *p* value	Target Gene ID’s
Complement	9	37	0.243	1.414	1.11E-06	C3, C1QA, C1QB, C1QC, C1S, CFD, CFH, ITGB2, SERPING1
Cholesterol Biosynthesis	7	28	0.250		2.63E-05	CYP51A1, HMGCR, HMGCS1, HSD17B7, IDI1, LSS, SC5D
iCOS-iCOSL signaling T helper cell	10	108	0.093		6.64E-04	CAMK2B, CD3G, FCER1G, HLA-DQA1, HLA-DQB1, HLA-DRA, HLA-DRB5, PDPK1, PLEKHA4, PTPRC
Cholesterol Biosynthesis I	4	13	0.308		1.84E-03	CYP51A1, HSD17B7, LSS, SC5D
Cholesterol Biosynthesis II (via 24,25-dihydrolanosterol)	4	13	0.308		1.84E-03	CYP51A1, HSD17B7, LSS, SC5D
Cholesterol Biosynthesis II (via desmosterol)	4	13	0.308		1.84E-03	CYP51A1, HSD17B7, LSS, SC5D
Ca-induced T cell Apoptosis	7	64	0.109		2.40E-03	CD3G, FCER1G, HLA-DQA1, HLA-DQB1, HLA-DRA, HLA-DRB5, PRKCE
OX40-signaling	8	89	0.090		2.40E-03	CD3G, FCER1G, HLA-DQA1, HLA-DQB1, HLA-DRA, HLA-DRB5, RT1-EC2, TNFSF4
NFAT regulation of immune response	11	171	0.064	1.89	2.40E-03	BLNK, CD3G, FCER1G, FCGR2B, FCGR3A/FCGR3B, GNAS, GSK3B, HLA-DQA1, HLA-DQB1, HLA-DRA, HLA-DRB5
B Cell development	5	33	0.152		4.35E-03	HLA-DQA1, HLA-DQB1, HLA-DRA, HLA-DRB5, PTPRC
Nur77 signaling in T cells	6	57	0.105		6.70E-03	CD3G, FCER1G, HLA-DQA1, HLA-DQB1, HLA-DRA, HLA-DRB5
Cd28 signaling in T Helper Cells	8	118	0.068		1.15E-02	CD3G, FCER1G, HLA-DQA1, HLA-DQB1, HLA-DRA, HLA-DRB5, PDPK1, PTPRC
PKC-τ signaling in T cells	8	118	0.068	1.414	1.15E-02	CAMK2B, CD3G, FCER1G, HLA-DQA1, HLA-DQB1, HLA-DRA, HLA-DRB5, MAP3K8

The differentially expressed annotated genes (target gene set) were placed in the contexts of canonical biological pathways using the IPA software and its curated database. Ratio = target genes/pathway genes. *P* values were calculated using a modified Fisher test that corrects for multiple hypothesis testing. Significant Z-score indicate overall pathway modulation (+) for up (-) for down

Among the remaining 12 pathways, four involved cholesterol metabolism, while nearly all remaining pathways were related to the T-helper cell/antigen presenting cell (APC) or T cell signaling (Table 3). These other pathways overlapped with common immune cell markers including Cd3g, Ptprc, Fcer1g, and MHC-I and MHC-II related genes. This is well illustrated in the O× 40-signaling pathway, in which APC, such as microglia, present antigen in the context of MHC and O× 40 receptor ligand to effector T cells, leading to activation of Nfkb, cJun and PI3K/PKB pathways [24].

A number of mechanistic regulatory networks were also populated by the differentially expressed gene set (Table 4), including pro-inflammatory mechanistic networks, IFNG, LPS and TNF. Additionally the inosine network, an immunomodulatory adenosine metabolite, and vancomycin, a neurotoxic antibiotic, were also significantly represented. IFNG, the most well populated network contained the highest density of predicted interactions that correlated to actual changes in target gene expression. This 132-member network, featuring 19 regulatory nodes, was populated by 59 of the differentially expressed age-related genes (50% of the total, *p* = 1.24 × 10^−16^). The entire network was activated in middle age with an overall activation Z-score = 2.711. Differentially expressed genes showed large numbers of regulatory inputs including Atf3, Anxa1, Postn, Cp, Cebpd, Fcgr2b, S100a8, C3, Col2a1, Map3k8 And Ppargc1b. Major regulatory nodes include Ctnnb1, Nfkbia, Nfkb, Smad3, Sp1 and Il4.

**Table 4:** Populated upstream regulatory networks

Upstream Regulator	Molecule Type	*p*-value of overlap	Target molecules in dataset	Mechanistic Network
IFNG	Pro-inflammatory cytokine produced mainly by activated T cells	1.33E-16	ABCA1,AIF1,ASS1,AVPR1A,BLNK,C1QA,C1QB,C1QC,C3,C4A/C4B,CCND2,CD163,CD74,CDH22,Cebpd,CP,CTGF,FAM107A,FCER1G,FCGR2B,FCGR3A,HLA-DQA1,HLADRA,HMGCR,HMGCS1,IDI1,IFITM1,IGF1,ITGB2,LGALS3,MAP3K8,MERTK,MT1E,PHACTR1,PLEK,PPARGC1B,RT1EC2,SERPING1,SLC6A6,SLC9A3,STAT6,TIMP1,TLR7,TREM2,TYROBP	CEBPA,FOXO3,Hdac,IFNG,IL1B,IL4,LDL,NFkBcomplex,PPARG,RUNX2,SP1,SP3,STAT1,STAT3,STAT5a/b,STAT6,TNF
Inosine	Metabolite with immunomodulatory effects	4.15E-16	AIF1,ANXA1,C1QA,C1QB,C1QC,C1S,C3,C4A/B,CFD,MS4a6b,MT1E,SERPING1,TIMP1	
LPS	Major component of outer membrane of Gram-negative bacteria	2.92E-14	ABCA1,ANXA1,ANXA3,ASAP1,ASS1,AVPR1A,BMP2,C3,CCND2,CD163,CD37,CD53,CD74,CFD,CP,CSF3R,CTGF,DDX6,DIO2,EMR1,FCER1G,FCGR2B,Gp49a/Lilrb4,HLADQA1,IFITM1,IGF1,ITGB2,LGALS3,MAFF,MAP3K8,MERTK,MMP14,MT1E,MT1H,PDE4B,PLD4,PLEK,PPARGC1B,PYCARD,SLC9A3,SLFN13,STAT6,TIMP1,TLR7,TNFSF4,TREM2,TYROBP,VEZF1	CEBPA,FOXO3,HDAC,IFNG,IL1B,IL4,IL6,NCOA1,NFkB (complex),PPARG,RUNX2,SP1,SP3,STAT1,STAT3,STAT4,STAT5a/b,STAT6,TNF,lipopolysaccharide
DYSF	Ca2+ sensor protein involved in synaptic vesicle fusion	4.36E-12	ABCA1,ANXA1,C1QB,CD53,CD74,CFD,FCGR2B,GP49a/Lilrb4,HLA-DQA1,LGALS3,LYZ1/LYZ2,TIMP1	
Vancomycin	Antibiotic that exhibits significant neurotoxicity	8.00E-12	ANXA1,C3,C4A/C4B,CP,FMO2,GPNMB,LGALS3,MS4a6b,S100A11,SCD,TIMP1,TMEM173	
TNF	Pro-inflammatory cytokine; In the CNS, derived mainly from activated microglia	2.31E-11	ABCA1,ANXA1,ASS1,AVPR1A,BIRC7,BMP2,C3,CCND2,CD163,Cebpd,CFD,CP,CTGF,FCER1G,FCGR2B,HERC1,HLADRA,HMGCR,HSD17B7,IFITM1,IGF1,ITGB2,LGALS3,LRG1,LSS,MAFF,MAP3K8,MMP14,MT1E,MT1H,PDE4B,PER2,POSTN,PPARGC1B,PYCARD,RGS4,SCD,SLC9A3,TIMP1,TLR7,TNR,TREM2	CEBPA,CTNNB1,FOXO3,IFNG,IL1B,IL4,NFkB complex),PI3K complex,SP1,SP3,STAT1,STAT3,STAT5a/b,STAT6,TNF,estrogen receptor

Members of the annotated differential gene set (353) were fit to regulatory networks in the IPA data base. *P* values are the probability that the observed overlap occurred by chance alone

The transcriptional mechanistic regulatory networks were also well represented. Ctnnb1, part of the canonical WNT pathway, is a co-activator of TCF/LEF family transcription factors, which up-modulates expression of WNT-responsive genes [25]. Out of 124 members, this network contained 36 of the differentially expressed, age-related genes (*p* = 3.96 × 10^−12^). Major regulatory nodes included Ctnnb1, Pge2, Smad3, Sp1, and Jun. Differentially expressed genes having multiple regulatory inputs included Igfbp5, Bmp2, Sox2, Ccnd2, Gja1, Ctgf, Mmp14 and Cxcl9. Overall this transcriptional network was significantly inhibited in middle age (Z-score = -2.377) which could explain the down-modulation WNT-responsive genes: Ccnd2, Gja1, Igf1, Ppap2b, Sox2, and Tcf7l2. On the other hand, two Ctnnb1 genes, that are also STAT regulated, were up modulated in older LSC, as are Stat6 and a synergistic co-activator Cepbd.

Bio-functional networks were also explored. The top functional populated networks were systemic autoimmune syndrome and activated immune cell adhesion and migration (Network 01). This network had the highest consistency score (10.104) with a total of 21 nodes and 5 regulators and 12 differentially expressed LSC target genes that formed the core of the immune cell regulatory network, under the control of Tmg2, Ifnαr, Gata4, and/or Hbb-b1. Strong relationships included activation of immune cell adhesion and leukocyte chemotaxis involving S100a8, C3 and Cd74 expression, and migration of antigen presenting cells associated with expression of Tyrobp, Il16, S100a4 and C3. All functional network members are listed in Additional files 4, 5, 6 and 7.

Multiple other functional networks overlapped Bio-functional Network 01. All were related to immune cell function, adhesion and migration. Network 02 had a consistency score of 6.640, with 19 nodes, 5 regulators and 12 targets. Interactions stemming from up-modulation by Nfkb complex of C3, Plau, Tnsf4, Map3k8, and Ctgf expression were associated with activation of macrophage migration; whereas, increased expression of C1qa, S100a8, Cd74, Lgals3, C3 and Plau, under Hrg, Tgm2, Nfκbia, or Nfκb complex control, was associated with immune cell adhesion. Functional networks 03 and 04 (consistency scores of 5.078 and 3.618, respectively) overlapped. Network 03, which had 21 nodes, 6 regulators, and 14 target genes, featured Ifng and/or Ifnαr driving expression of Cxcl9, Cd74, C3, S100a8, Stat6 and Aif1 expression associated with leukocyte chemotaxis; whereas Network 04, which had 16 nodes, 3 regulators and 11 targets, featured Tlr9, Tlr3, or transcriptional regulator, Ehf, modulating expression of C3, Map3k8, S100a8, Timp1 and, Abca1 that was associated with activation of myeloid lineage cell migration. The consistency of each network and the overlaps indicate a strong association of these members of the age-related gene expression set with immune cell adhesion and migration.

We had previously reported that most microglia of healthy middle-aged rat LSC exhibited a activated morphology compared to young adults [14]. Here we find that nearly 10% of the differential gene set was associated entirely or primarily with changing patterns of microglial gene expression (Table 5). Approximately 75% of these transcripts were related to microglial activation [26, 27], and substantially overlapped with neuropathic pain-modulated spinal cord genes that were described in previous transcriptomic studies [28, 29]. In addition to microglial associated genes, other neuropathic pain-related transcripts were differentially expressed, yielding a total of 43 such genes or ~12% of the entire age-related LSC transcriptome (annotated).

**Table 5:** Microglial and neuropathic pain-related gene expression

Gene	M/Y	Microglial	Pain	Function
ABCA1	1.62	Y	Y	Transporter of cholesterol and other lipids
AIF1	1.53	Y	Y	Forms complexes with L-fimbrin in membrane ruffles and phagocytic cups; may modulate actin reorganization, facilitates cell migration and phagocytosis
ANXA3	1.55	Y	Y	Annexin family member; up-regulated following tissue injury
ATF3	1.73	Y	Y	Complexes with other transcriptional regulators; up-regulated in response to injury; negative regulator of TLR signaling; suppresses innate immune response
BLNK	1.68	Y		Cytoplasmic linker protein important in B cell development
C1QA	1.61	Y	Y	Part of C1q complex; initial component of classic pathway
C1QB	1.59	Y	Y	Part of C1q complex
C1QC	1.52	Y	Y	Part of C1q complex
C4A	1.98	Y	Y	Cleaved by C1S; combines with C2 cleavage product forms C4b2b complex
CAMK2B	0.64	Y	Y	Ca2+/calmodulin-dependent protein kinase
CD163	1.59	Y		M2 state microglial marker (aka, ED2); Alt priming gene
CD37	1.54	Y	Y	Interacts with dectin; regulates PAMP induced IL6 production; microglial sensome
CD53	1.71	Y	Y	Contributes to transduction of CD2-generated signals in T cells
CD74	1.85	Y	Y	Invariant MHCII component; processes receptor bound PAMP’S and DAMP’s for presentation to T cells
CSF3R	1.59	Y	Y	Binds GCSF, which increases M2 state marker expression, and inhibits pro-inflammatory cytokine expression, while promoting neurotrophic factor expression.
CXCL9	1.68	Y	Y	Ligand for Cxcr3 receptor, activated by TLR ligands and pro-inflammatory cytokines; appears to regulate T cell migration
Egr2	1.96	Y		Transcription factor; Alt priming gene
EMR1	1.81	Y	Y	Member of adhesion GPCR receptors; required to activate CD8+ regulatory T cells
FAM105A	2.08	Y	Y	
FCER1G	1.74	Y	Y	High affinity Fc epsilon receptor; binds TREM2; involved in response to apoptotic debris, immune complexes through C1q complex
FCGR2B	2.89	Y	Y	Fc receptor, IgG, low affinity; binds Cxcl7 (CD32B); inhibitory
FCGR3A	1.52	Y	Y	Aka CD16a; similar to FCGR2B
FCRLS	2.78	Y		Fc immunoglobulin receptor; part of TGFb1 microglial signature response
GOLM1	1.53	Y		Assists transport of cargo through Golgi apparatus; AD risk gene
IGF1	0.65	Y		Neurotrophic factor; increased by IL4/MCSF; M2 phenotype and Alt priming gene
IGFBP6	1.53	Y	Y	Binds IGF’s and modulates their growth factor effects
ITGB2	1.73	Y	Y	CD18; integrin beta chain beta 2; involved in leukocyte adhesion
LGALS3	2.44	Y		Galectin family; inflammatory factor (MAC2); microglial marker; Alt priming gene
PTPRC	1.84	Y	Y	Protein tyrosine phosphatase; aka CD45; enriched in microglia; opposes microglial activation
PYCARD	1.50	Y	Y	Mediator of apoptosis and inflammation; adapter for inflammasome assembly
TLR7	1.84	Y	Y	TLR (nucleotide sensing); signals via MyD88 pathway; can modulate other TLR’s
TMEM173	1.52	Y	Y	Facilitates innate immune signaling; promotes expression of IFN-alpha and IFN-beta;
TREM2	1.74	Y		Forms signaling complex with TYROBP; M2 state and sensome gene; promotes microglial expansion; up-regulated by IL4; critical for normal phagocytosis
TYROBP	1.82	Y	Y	Forms signaling complex with TREM2; activates microglia; required for synaptic pruning

The annotated differentially expressed gene set (353) was compared to previously published consensus genes specific for microglia. Those relevant to establishment of neuropathic pain are indicated

## Discussion

We have previously published cytokine and immune marker gene expression and immunohistochemical evidence that a para-inflammatory state develops in the LSC by middle age [14]. We reported that microglia of healthy middle-aged rat LSC showed a predominately activated phenotype, whereas astrocyte morphology and GFAP protein levels indicated quiescence [14]. Results also showed that Cd2, Cd3e, Cd68, Cd45, Tnf-α, Il6, Ccl2, Atf3 and Tgfβ1 mRNA levels were substantially elevated. Here we extend that study and report that the transcriptomic changes associated with middle age are dominated by up-modulation of the innate immune system, including complement, TLR signaling, T-cell/APC interface, microglial priming, and M1 and M2 activation states. A number of other immune regulatory pathways were significantly up modulated including NFAT transcriptional pathways, important for neuronal excitation-transcription coupling and neurotrophin signaling [30], and the T-cell/PKC-Tau pathway. Components of other pathways involved in the regulatory interface between APC’s and T-cells were similarly affected with increased expression of components in the OX40, icos-icosL, Nur77, and Cd28 signaling. Many immune regulatory networks were affected by age. Three of the top six networks control or activate central inflammatory responses involving glia and T-cells, which fit well the view that the innate immune system in the healthy LSC in middle age is in a state of incipient activation.

In a detailed study of the mouse life span, 127 genes were identified as aging-related in brain [31]. Eighteen genes associated with middle age in the spinal cord were similarly modulated in in the mouse brain data set, with 8 of these expressed in microglia. A recent combined transcriptomic and proteomic study of aging brain and liver also reported up-modulation of genes associated with antigen processing and presentation and immune system responses in senescent rat brains compared to those of young adults (two of the top three functional classifications) [32]. Thirty of the same genes reported here for middle-aged LSC were identified in the 609 differentially expressed genes in senescent rat brain [32], and 12 of these common genes were related to microglial function and/or establishment of neuropathic pain, including a number of complement components.

While our results are consistent with those previously reported for the senescent brain and other aging tissues, few of the genes we report are represented in the differential gene set previously reported for middle-aged and young adult mouse spinal cord [33]. Only 7, Acbd3, Elovl6, Jam2, Matn2, Nedd4, Pank3, Phactr1, and Ryr2 were found in both differential gene sets. Of these, the directions of modulations in 5 (Elovl6, Jam2, Nedd4, Phactr1, Ryr2) were not in agreement. The reasons underlying the lack of consistency with our study is unclear, though species differences may have contributed, as well as the many variables explored in the previous work (age, gender, tissue), that could have reduced the power to reliably identify differentially expressed genes in middle-aged mouse spinal cord. In two previous transcriptomic studies of middle-aged brain reported by Loerch and co-workers [34] and by Wood and others [35], only 3 (Csnk2a1, Hmgcr, Sgtb) and 4 (RT1-Ba, RT1-Bb, RT1-Da, Sema3b), respectively, were in common with the differential set reported here, suggesting the changes in middle-aged spinal cord we report here may be unique to the spinal cord. It is also possible that dissimilar platforms could have contributed the lack of overlap.

Up-modulation of the complement pathway activation is one of the most common age [36] or neuropathic pain related changes reported for the CNS [37]. A detailed study of complement component expression in aging mouse forebrain also reported significant increases in C1q and C3 transcript levels [38], similar to those reported here. Unlike the LSC, C4 transcript levels were not significantly elevated until 24 months in the mouse forebrain. We also measured significant increases (by qPCR) in transcripts encoding Cd11b or Cr3 (Table 2), a common microglial activation marker and the receptor for C3 complement, which has been reported to increase in a variety of different CNS pathologies [39–41]. While many activating components were up modulated in the middle-aged LSC, so were the transcripts of several counter regulatory components encoding proteins that block formation of C1q protein or increase the degradation of C3a, suggesting that complement activation in middle age could be suppressed.

A substantial fraction of the age-associated LSC transcriptome (Table 5) belongs to the microglial sensome [26, 27]. Most identified here are related to M1 or M2 activation states. Cd74 (MHCII invariant chain) encodes a marker of microglial M1 activation [42–44] and is increased in the brains of aging rodents [27] and non-human primates [45]. MHCII complex, identified in the canonical pathways up modulated in LSC, is key on APC/T-cell interface [24]. Consistent with M1 state activation were the increases in transcripts encoding pro-inflammatory chemokine Cxcl9, Cd16 (Fcgr2a, Fcgr3b), Cd32 (Fcgr2b) and Cd45 (Ptprc) [42–44]. On the other hand, many M2 sub-state activation markers and associated microglial transcripts were also up modulated including Cd163, Trem2, Tyrobp, Lgals3, and Csfr3 [42–44]. Some transcripts identified in the differential gene set are also implicated in activating microglial phagocytosis; these include Trem2, Tyrobp, Fcer1g, Cd32, and Aif1 [44, 46]. Enhanced phagocytosis is associated with M2 activation and with beneficial anti-inflammatory effects and augmented recovery/resolution or lower risk of neurodegenerative changes [42, 46]. Overall the evidence supports the evolution of multiple different microglia activation states during aging in the spinal cord, any one of which could alter the innate immune response to injury or infection, and the trajectory of recovery or resolution.

We also report here a general down modulation of transcripts encoding enzymes involved in cholesterol metabolism including Hmgrc, Hmgcs1, Cyp51, Lss, Sc5d and Scd1; whereas the cholesterol transporter, Abca1, was up-modulated. Implications for spinal cord cholesterol, however, are unclear. Comparable changes have been previously reported, in a study of aging cervical spinal cord, that were associated with perturbation of cholesterol homeostasis (increased white matter cholesterol ester concentrations) and inflammatory activation in the cervical spinal cord [47]. Age-related changes in CNS cholesterol metabolism are believed to play a key role in the development of some forms of Alzheimer’s disease and other degenerative disorders [48].

Overall, these results provide further support for “inflammaging” of the LSC by middle age in rat, which has been amply demonstrated in other senescent tissues of many species, including humans [6]. Such emerging inflammatory changes in the LSC may be most relevant to human spinal cord neuropathies, particularly the increased risk of chronic pain, which is strongly associated with middle age, rather than senescence [8, 10–13, 49, 50]. The spinal cord is the primary site of first order nociceptive signal processing; the first relay in transmission to higher centers; and is the origin of hyperalgesia and spontaneous pain, all driven by activated microglia, astrocytes and the innate immune system [51–53]. Nonetheless, our study did not investigate changes in the aging rat proteome, nor did we explore here any alterations in the responses to injury or to degeneration of the somatosensory system; therefore these potential implications remain speculative. Furthermore, to test whether our results are relevant to the risks of chronic pain or the failures of current treatments in middle-aged humans will require investigations of post-mortem spinal cords.

## Conclusions

Transcriptomic analysis of healthy middle-aged LSC demonstrated up-modulation of complement pathway, microglial priming and activation, and T cell/antigen-presenting cell communication. Taken together with previous work, the results support our conclusion that an incipient or para-inflammatory state develops in the LSC in healthy middle-aged adults.

## Additional files

Additional file 1: Primer sequences used in the qPCR measurements. (PDF 34 kb)

Additional file 2: Up and down modulated genes. Description: all genes significantly up or down modulated in LSC comparing young to middle-aged LSC. (XLSX 445 kb)

Additional file 3: Differentially expressed gene set. Description: all annotated genes included in the differentially expressed gene set that were included in the pathway analysis. (XLSX 38 kb)

Additional file 4: Bio-functional Network 01. Description: differentially expressed gene set: pink to red (intensity indicates degree of up-modulation), while green denotes down-modulation). Colors of upstream regulators and downstream biological processes are related to predicted activation states with orange indicating activation and blue denoting inhibition. Lines connecting nodes are orange when leading to activation, blue when inhibition is predicted, and yellow if relationships are not consistent with the downstream node state shown. Gray lines denote lack of evidence to form a prediction. Solid lines show direct relations and dashed indirect. Blunted ends indicate expected inhibition if upstream node is activated. (TIFF 2369 kb)

Additional file 5: Bio-functional Network 02. Description: as for Additional file 4. (TIFF 2216 kb)

Additional file 6: Bio-functional Network 03. Description: as for Additional file 4. (TIFF 2215 kb)

Additional file 7: Bio-functional Network 04. Description: as for Additional file 4. (TIFF 2311 kb)

## Abbreviations

APC: Antigen presenting cell
CNS: Central nervous system
LSC: Lumbar spinal cord
qPCR: Quantitative polymerase chain reaction
