# Supplementary material for: Transcriptomic evidence of a para-inflammatory state in the middle aged lumbar spinal cord
Source: Immun Ageing. 2017 Apr 13;14:9. doi: 10.1186/s12979-017-0091-6 (PMC5390443; doi:10.1186/s12979-017-0091-6)
Supplement: Supplementary file 1 — Primer sequences used in the qPCR measurements. (PDF 34 kb) [file 12979_2017_91_MOESM1_ESM.pdf]

SUPPLEMENTARY TABLE 3: Primer sequences used in the qPCR measurements

**Gene**	**Forward**	**Reverse**
Lgals	5’-CCCCGACTGGACCACTGA-3’	5’-GAGTGATACTGTTTGCGTTGGG-3’
Fcgr2b	5’-ACGCTAAATGTCACAGAGCCC-3’	5’-CGCGTCCTTCACAGTGGT-3’
Gpnmb	5’-TGCAAAGGGGCCACTCC-3’	5’-GGACAGGAGGCACAGCTC-3’
C3	5’-GCGGTACTACCAGACCATCG-3’	5’-GCCGATCTTCAAGGGGACAA-3’
Atf3	5’-TACTGCGTTGTCCCACTCTG-3’	5’-GGCCAGCTAGGTCATCTGAG-3’
Ptprc	5’-CCACGGGTATTCAGCAAGTT-3’	5’-AGAGAGCTCCACACGGTTGT-3’
Cd163	5’-AGCCATCGGAATCATCAGAC-3’	5’-GCTCACAGCCACAACAAAGA-3’
Nrg1	5’-GGGACCAGCCATCTCATAAA-3’	5’-ATCTTGACGGGTTTGACAGG-3’
Cd11b	5’-CCAGTGTGACATCCCTTCCT-3’	5’-AGTGCTCACAAGCAGGAGGT-3’
Gapdh	5’-CAGGGCTGCCTTCTCTTGTG-3’	5’-AACTTGCCGTGGGTAGAGTC-3’
