# Supplementary figures and images for: Transcriptomic evidence of a para-inflammatory state in the middle aged lumbar spinal cord
Source: Immun Ageing. 2017 Apr 13;14:9. doi: 10.1186/s12979-017-0091-6 (PMC5390443; doi:10.1186/s12979-017-0091-6)

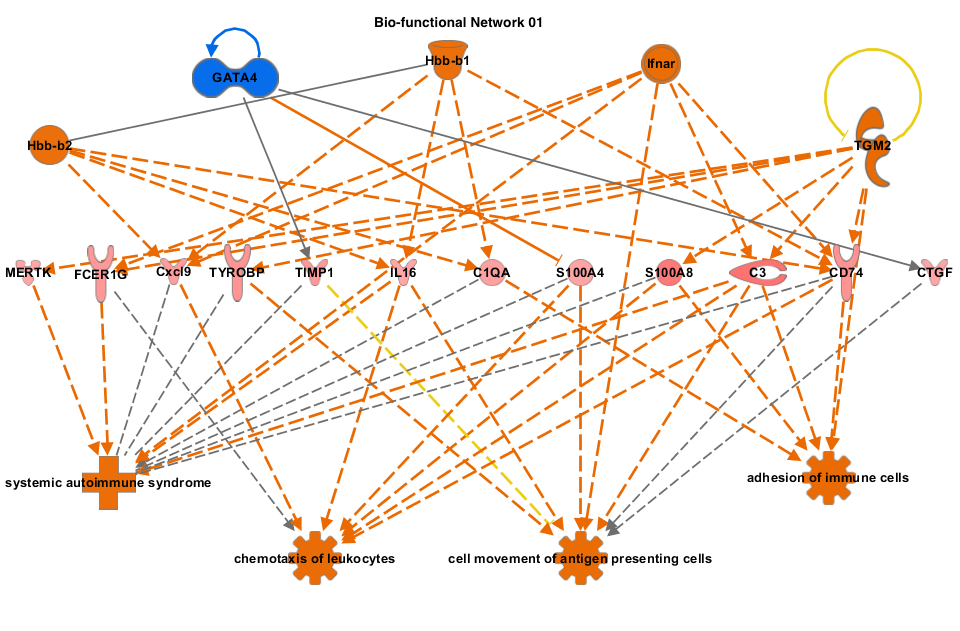

Supplement: Supplementary file 4 — Bio-functional Network 01. Description: differentially expressed gene set: pink to red (intensity indicates degree of up-modulation), while green denotes down-modulation). Colors of upstream regulators and downstream biological processes are related to predicted activation states with orange indicating activation and blue denoting inhibition. Lines connecting nodes are orange when leading to activation, blue when inhibition is predicted, and yellow if relationships are not consistent with the downstream node state shown. Gray lines denote lack of evidence to form a prediction. Solid lines show direct relations and dashed indirect. Blunted ends indicate expected inhibition if upstream node is activated. (TIFF 2369 kb) [file 12979_2017_91_MOESM4_ESM.tiff]

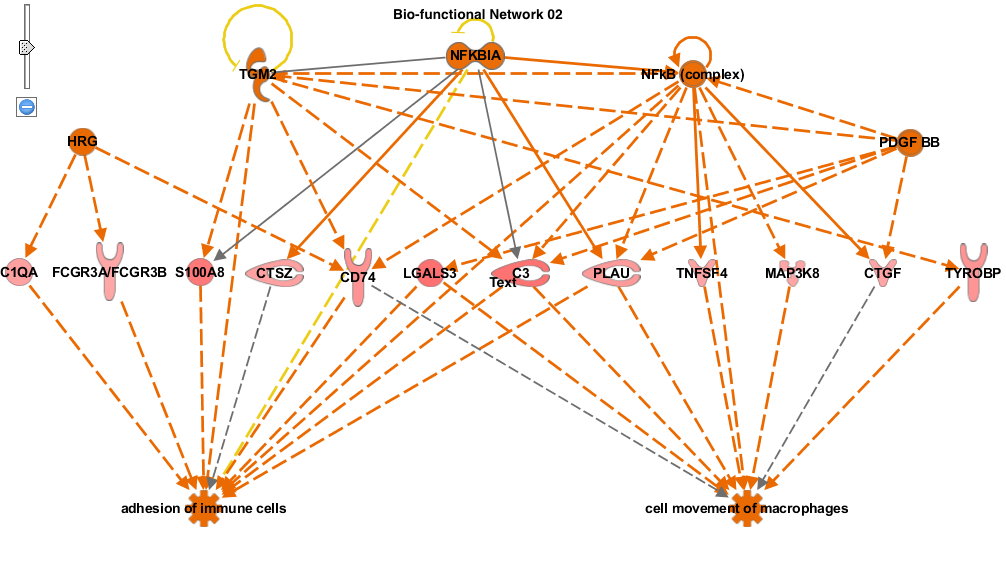

Supplement: Supplementary file 5 — Bio-functional Network 02. Description: as for Additional file 4. (TIFF 2216 kb) [file 12979_2017_91_MOESM5_ESM.tiff]

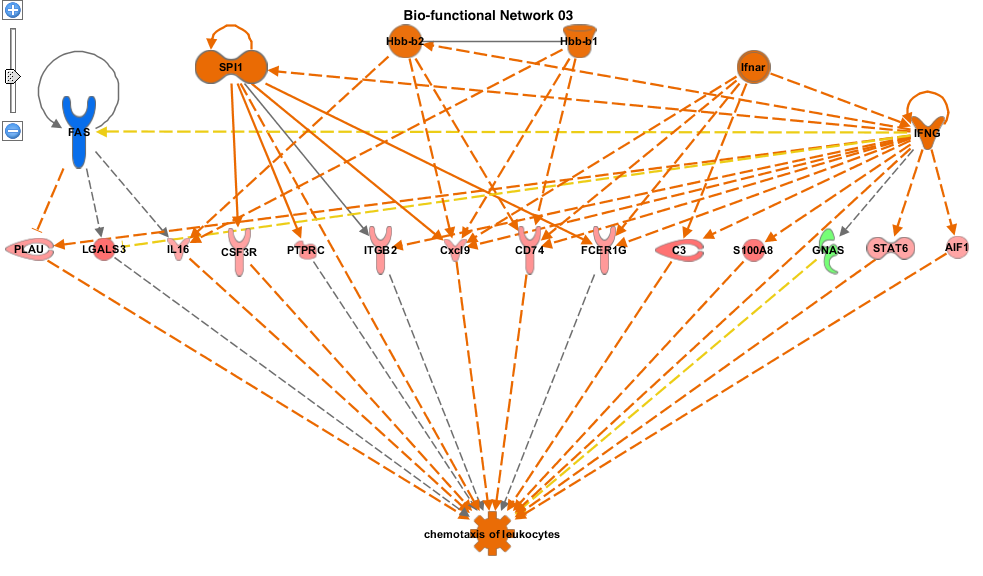

Supplement: Supplementary file 6 — Bio-functional Network 03. Description: as for Additional file 4. (TIFF 2215 kb) [file 12979_2017_91_MOESM6_ESM.tiff]

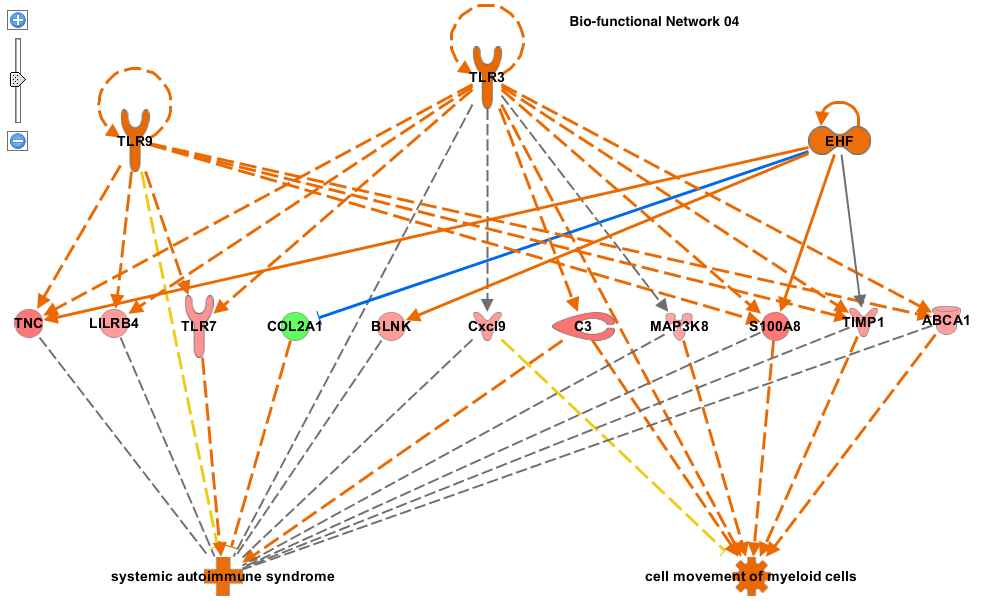

Supplement: Supplementary file 7 — Bio-functional Network 04. Description: as for Additional file 4. (TIFF 2311 kb) [file 12979_2017_91_MOESM7_ESM.tiff]
